# Risk factors for elevated serum colorectal cancer markers in patients with type 2 diabetes mellitus 

**DOI:** 10.1515/biol-2020-0030

**Published:** 2020-05-26

**Authors:** Jingjing Guo, Bin Wang, Weikai Hou, Kun Ma

**Affiliations:** Department of Health, Jinan Central Hospital Affiliated to Shandong First Medical University, Jinan 250013, China; Department of Endocrinology, Qilu Hospital of Shandong University, No. 105, Jiefang Road, Jinan 250012, China; Department of Pediatrics, The First Affiliated Hospital of Shandong First Medical University, No. 16766, Jing Shi Road, Jinan 250014, China

**Keywords:** colorectal cancer, carbohydrate antigen, carbohydrate antigen, methylated septin9, body mass index, vitamin D, glycated hemoglobin A1c, triglyceride

## Abstract

The study aims to examine the risk factors for increased colorectal cancer (CRC) markers in patients with type 2 diabetes mellitus (T2DM). The 229 patients retrospectively reviewed were categorized into two groups: CRC tumor marker-positive and -negative groups. Patients who tested positive for all three of the following CRC markers were included in the CRC tumor marker-positive group: serum carcinoembryonic antigen, carbohydrate antigen 19-9 and septin9 methylation. Univariate analysis revealed that most CRC marker-positive patients had higher age, a family history of CRC, history of smoking and alcohol intake, high body mass index (BMI; overweight), longer history of T2DM, worse diabetes control (with high glycated hemoglobin A1c [HbA1c]), lower level of serum vitamin D (VD), high-density lipoprotein cholesterol and higher level of total cholesterol and triglyceride (TG). Logistic regression analysis showed that BMI, VD, HbA1c and TG were independent predictors of CRC marker-positive status (OR, 95% confidence intervals and *P* values were 1.912 [1.346–2.716], <0.001; 0.773 [0.633–0.943], 0.011; 9.082 [3.52–23.433], <0.001; and 11.597 [3.267–41.164], <0.001, respectively). In this retrospective study, high BMI, HbA1c and TG as well as low level of VD were correlated with CRC tumor marker-positive status in T2DM patients. Patients with these risk factors may benefit from more frequent screening for CRC tumor markers.

## 1 Introduction

The prevalence of type 2 diabetes mellitus (T2DM) has shown a steady increase alongside the upward socioeconomic mobility and the general improvement in the standards of living. Previous studies have shown that patients with T2DM are at a higher risk of developing malignant tumor compared to nondiabetic individuals [1]. Epidemiological studies have shown that diabetes is an independent risk factor for colorectal cancer (CRC); the risk of CRC in T2DM patients is approximately 1.30 times higher than that of nondiabetic patients [2]. For hepatocellular carcinoma, pancreatic cancer and renal carcinoma, this number is 1.26, 2.81 and 1.61 in males and 1.53, 3.62 and 1.71 in females, respectively [3]. The mechanism of the association between T2DM and the risk of cancer is complex and multifaceted. Exogenous insulin promotes overexpression of insulin-like receptors, thereby promoting mitosis of cancer cells [4]. T2DM induces a certain degree of immune dysfunction. In addition, the role of vitamin D (VD) in the process of tumorigenesis has increasingly been acknowledged in recent years. A meta-analysis of studies (combined study population of 17,664 subjects) revealed that patients with T2DM, especially those with diabetic nephropathy, often have decreased serum VD levels [5]; and decreased serum VD levels were shown to increase the risk of malignant tumors [6]. Therefore, a variety of factors appear to influence the incidence of malignant tumors in patients with T2DM.

CRC is a malignant tumor that predominantly occurs in middle-aged and elderly people [7]. Patients typically exhibit no obvious clinical symptoms in the early stages of the disease. Screening of serum markers is an important tool for early diagnosis of CRC [8]. At present, the more commonly used serum markers include carbohydrate antigen (CA) 19-9, CA50, CA125 and the carcinoembryonic antigen (CEA). CA19-9 has a high positive predictive value for digestive system carcinomas, such as CRC, pancreatic cancer, gallbladder cancer, cholangiocarcinoma and gastric cancer. In addition, a mild increase in CA19-9 occurs in noncancerous diseases, such as chronic hepatitis, cirrhosis and pancreatitis [9,10]. CA50 is not expressed or has a low expression in normal tissues; however, the serum levels increase significantly upon malignant transformation of cells. CA50 is a broad-spectrum tumor marker with poor specificity for diagnosis of cancers [10]. CA125 is a sensitive marker for the diagnosis of epithelial ovarian tumors, but its level is often increased in patients with CRC; therefore, it has a poor specificity for CRC [10,11]. CEA is regarded as a more specific marker of CRC. However, recent clinical studies have shown that CEA is also highly expressed in breast, gastric and pancreatic cancers [12]. *Septin9* is a gene that was shown to be directly related to cancer occurrence. Methylation of the CpG islands in the 5′-region of the *Septin9* gene has been implicated in the development of CRC. The incidence of hypermethylation in CRC tissue is up to 90% as against 10% in normal control tissues [13]. Therefore, the methylated septin9 (mSEPT9) is an important biological characteristic and serological marker for CRC. Methylated septin9DNA can be released from the necrotic or apoptotic tumor cells into the peripheral circulation in the early stages of CRC. Thus, detection of methylated septin9 in peripheral blood can be used for screening, early diagnosis, treatment evaluation and dynamic monitoring of CRC [14,15].

Although detection of serum markers cannot be used to confirm CRC diagnosis due to its poor specificity, it is still the most important means of CRC screening in China because of its good sensitivity, simple operation and relatively low price [16]. Because of the great correlation between T2DM and CRC, we infer that compared with the general population, the T2DM population may have higher serum levels of CRC markers and hence need more frequent detection. The aim of this study is to examine the risk factors associated with elevated serum markers of CRC in patients with T2DM. Our findings may help identify patients who may benefit from more frequent assessment of tumor marker levels.

## 2 Materials and methods

### 2.1 Study design

This was a retrospective case–control study. Adult patients (age >18 years) who were diagnosed with T2DM and treated at the Department of Health and Endocrinology of the Jinan Central Hospital between January 2016 and November 2017 were eligible for inclusion in this study. The control subjects were all healthy individuals who underwent a medical examination at the Jinan Central Hospital during the same time.


**Informed consent:** Informed consent has been obtained from all individuals included in this study.
**Ethical approval:** The research related to human use has been complied with all the relevant national regulations and institutional policies and also in accordance with the tenets of the Declaration of Helsinki, and the study has been approved by the ethics committee of the Jinan Central Hospital affiliated to the Shandong University.

### 2.2 Participants and grouping

The diagnosis of T2DM was based on the diagnostic criteria issued by World Health Organization in 1999: presence of clinical symptoms of diabetes plus random blood glucose (BG) > 11.1 mmol/L or fasting BG > 7.0 mmol/L, or oral glucose tolerance test 2 h BG > 11.1 mmol/L. Asymptomatic patients were required to qualify the BG diagnostic standard twice within a 2-week period. The exclusion criteria were patients with acute life-threatening primary disease such as acute cardiovascular and cerebrovascular disease, infection, moderate to severe systematic chronic disease (such as hypertension, rheumatism and liver or renal dysfunction), chronic infection (such as tuberculosis), psychosis, cancer, and pregnant or lactating women.

The study population was grouped according to the level of serum markers. The guidelines of the Chinese Society of Clinical Oncology for diagnosis and treatment of CRC (2015) recommend detection of CEA and CA19-9 to support the diagnosis of CRC [17]; in addition, mSEPT9 is a relatively new and highly specific CRC tumor marker, which is now recommended by the Chinese Medical Association and the Chinese Anti-Cancer Association as a CRC screening biomarker [17]. Therefore, in this study, patients who tested positive for all three markers were included in the CRC marker-positive group and those who tested negative for all three markers were included in the CRC marker-negative group. Patients who tested positive for any one or two markers were excluded from this study.

### 2.3 Detection of CEA, CA 19-9 and mSEPT9

Serum levels of CEA and CA 19-9 were determined by fluorescent magnetic particle immunoassay kits (ST AIA-PACK CEA kit and ST AIA-PACK CA199 kit; Tosoh Hi-Tech Inc., Tokyo, Japan) on an AIA-2000 automatic immune analyzer (Tosoh Hi-Tech Inc.). The CEA level > 6 ng/mL and CA 19-9 level > 37 U/mL were considered positive. Peripheral blood mSEPT9 was detected by the probe-based real-time polymerase chain reaction (PCR) method, with the Septin9 Methylation Detection kit (Biochain Beijing Technology Co., Ltd, Beijing, China), according to the kit instructions. Briefly, DNA was extracted from peripheral blood. The methylated DNA was directly extracted by the magnetic bead adsorption, and the nonmethylated DNA was converted by the deamination reaction. The real-time PCR was performed on the ABI7500 real-time thermocycler (Applied Biosystems, Foster City, USA). The reaction conditions were as follows: 94°C for 20 min; 62°C for 5 s, 55.5°C for 35 s, 93°C for 30 s, for totally 45 cycles; followed by 40°C for 5 s. The results were interpreted using the ABI 7500 Fast PCR software (Applied Biosystems). Experiment was performed in triplicates.

### 2.4 Variables

Data pertaining to the following variables were included in the analysis: gender, age (years), duration of T2DM (years), family history of T2DM, family history of CRC, smoking, consumption of alcohol, body mass index (BMI, kg/m^2^), serum VD (ng/mL), glycated hemoglobin A1c (HbA1c, %), high-density lipoprotein cholesterol (HDL-C, mmol/L), low-density lipoprotein cholesterol (LDL-C, mmol/L), total cholesterol (Chol T, mmol/L) and triglycerides (TGs, mmol/L). Patients who smoked ≤20 cigarettes per week were considered as nonsmokers. Regarding alcohol consumption, men who consumed <140 g alcohol per week and women who consumed ≤70 g alcohol per week were considered as nonconsumers of alcohol. Subjects who had quit smoking or consuming alcohol for more than 5 years were considered as nonsmokers or nonconsumers of alcohol, respectively.

### 2.5 Statistical analysis

SPSS16.0 was used for statistical analysis. Qualitative variables were presented as frequencies and percentages. The distribution of quantitative variables was tested for normality using the Kolmogorov–Smirnov test. Quantitative variables with nonnormal distribution are presented as median (percentile 25, percentile 75), and quantitative variables with normal distribution are presented as mean ± SD. Analyses of factors that affected the CRC tumor marker status of patients with T2DM were performed as follows: first, a univariate logistic analysis was used for qualitative variables; an independent *t* test was used to assess the between-group differences with respect to normally distributed quantitative variables; and the Mann–Whitney test was used for nonnormally distributed quantitative variables. Variables that showed a significant association in the univariate analysis were included in the multivariate logistic regression model using a stepwise method to identify independent risk factors for elevated CRC tumor markers. *P* < 0.05 was considered indicative of statistically significant between-group difference.

## 3 Results

### 3.1 General data and univariate analysis of the participants

A total of 229 subjects with T2DM were enrolled in the study. Among them, 100 patients were categorized as CRC marker positive. Tables 1 and 2 show the general characteristics of patients and results of univariate analysis. Patients were grouped as CRC marker-positive when CEA level > 6 ng/mL, CA 19-9 level > 37 U/mL, and mSEPT9 was detected, and CRC marker-negative group when these markers were negative or with levels below threshold. The results of univariate analysis of qualitative variables are presented in Table 1, while those of quantitative variables are presented in Tables 2 and 3. The results showed that compared with the CRC marker-negative group, most patients in the CRC marker-positive group had a family history of CRC (Tables 1 and 2). Smoking and alcohol intake also showed a significant positive association with the CRC marker-positive group. Patients in the CRC marker-positive group exhibited higher age, were overweight (with high BMI), had a longer history of T2DM and exhibited worse diabetes control (with high HbA1c). Of note, the VD level in the CRC marker-positive group was significantly lower than that in the CRC marker-negative group. In addition, increased HDL-C level showed a negative correlation with the CRC marker-positive status, while increase in other blood lipid parameters showed a positive correlation with the CRC marker-positive status.

**Table 1:** Univariate analysis of qualitative variables

Factors		*n*	CRC markers		OR and 95% CI	*P* value
			Negative	Positive		
Gender	Male	107	56 (52.34)	51 (47.66)	1	
	Female	122	73 (59.84)	49 (40.16)	0.737 (0.436–1.245)	0.254

FH of T2DM	No	204	117 (57.35)	87 (42.65)	1	
	Yes	25	12 (48.00)	13 (52.00)	1.457 (0.634–3.349)	0.375

FH of CRC	No	194	122 (62.89)	72 (37.11)	1	
	Yes	35	7 (20.00)	28 (80.00)	6.778 (2.817–16.307)	<0.001

Smoking	No	160	114 (71.25)	46 (28.75)	1	
	Yes	69	15 (21.74)	54 (78.26)	8.922 (4.581–17.377)	<0.001

Alcoholic	No	167	119 (71.26)	48 (28.74)	1	
	Yes	62	10 (16.13)	52 (83.87)	12.892 (6.058–27.434)	<0.001

Total		229	129	100		

CRC: colorectal cancer; OR: odds ratio; CI: confidence interval; FH: family history; T2DM: type 2 diabetes mellitus.

*P* < 0.05 was considered statistically significant.

**Table 2:** Univariate analysis of quantitative variables

Factors	Descriptive statistic	Values	CRC markers		*t*/*Z* value	*P* value
			Negative	Positive		
Age (years)	Mean ± SD	62.45 ± 8.49	60.65 ± 8.16	64.78 ± 8.37	3.754	<0.001
BMI (kg/m^2^)	Mean ± SD	24.60 ± 2.71	22.92 ± 2.10	26.75 ± 1.69	14.894	<0.001
DT (years)	Median (P_25_, P_75_)	8 (6, 11)	6.5 (5, 8)	11 (8, 14)	8.974	<0.001
VD (ng/mL)	Median (P_25_, P_75_)	20.30 (16.30, 23.25)	22.4 (20.20, 24.25)	16.25 (13.53, 20.10)	9.004	<0.001
HbA1c (%)	Median (P_25_, P_75_)	8.50 (7.50, 9.55)	7.60 (7.10, 8.50)	9.70 (9.10, 11.28)	11.436	<0.001
HDL-C (mmol/L)	Median (P_25_, P_75_)	1.06 (0.96, 1.22)	1.14 (1.04, 1.23)	1.02 (0.85, 1.16)	5.016	<0.001
LDL-C (mmol/L)	Median (P_25_, P_75_)	3.25 (2.69, 3.69)	3.22 (2.65, 3.65)	3.43 (3.05, 4.03)	3.234	0.001
Chol T (mmol/L)	Median (P_25_, P_75_)	5.22 (4.46, 6.32)	4.58 (4.25, 5.38)	6.28 (5.22, 7.08)	7.645	<0.001
TC (mmol/L)	Median (P_25_, P_75_)	2.31 (1.60, 2.63)	1.69 (1.46, 2.45)	2.56 (2.31, 2.99)	8.057	<0.001
*N*			129	100		

CRC: colorectal cancer; BMI: body mass index; DT: duration of type 2 diabetes mellitus; VD: 25′ OH vitamin D3; HbA1c: glycated hemoglobin A1c; HDL-C: high-density lipoprotein cholesterol; LDL-C: low-density lipoprotein cholesterol; Chol T: total cholesterol; TC: triglyceride.

Normally distributed quantitative variables are presented as mean ± standard deviation (SD) and between-group differences were assessed using the grouped *t* test. Nonnormally distributed quantitative variables are presented as median (percentile 25, percentile 75) and analyzed using the Mann–Whitney test. *P* < 0.05 was considered statistically significant.

**Table 3:** Results of normal distribution test of quantitative variables

Variables	Age	BMI	DT	VD	HbA1c	HDL-C	LDL-C	Chol T	TC
*Z* value	0.763	1.203	2.198	1.374	1.632	1.695	1.336	2.152	1.348
*P* value	0.605	0.111	<0.001	0.046	0.010	0.006	0.056	<0.001	0.053

BMI: body mass index; DT: duration of type 2 diabetes mellitus; VD: 25′ OH vitamin D3; HbA1c: glycated hemoglobin A1c; HDL-C: high-density lipoprotein cholesterol; LDL-C: low-density lipoprotein cholesterol; Chol T: cholesterol total; TC: triglyceride.

The normality of distribution of quantitative variables was tested by the Kolmogorov–Smirnov test and *P* < 0.05 was considered statistically significant.

### 3.2 Screening independent factors for CRC marker positive group

All variables that exhibited a significant association with CRC marker status, including age, BMI, duration of T2DM, VD, HbA1c, HDL-C, LDL-C, Chol T and TG, were included in the logistic regression model. Simple logistic regression indicated that BMI, VD, HbA1c and TG were independent predictors of CRC marker-positive status. When logistic stepwise regression analysis was performed using the nine factors as independent variables (α to enter at 0.05 and remove at 0.10), the variables eventually left in the model were BMI, VD, HbA1c and TG (Table 4).

**Table 4:** Results of multivariate analysis

Factors		Simple logistic regression		Stepwise logistic regression	
		OR and 95% CI	*P* value	OR and 95% CI	*P* value
FH of CRC	No	1			
	Yes	1.624 (0.201–13.154)	0.650		
Smoking	No	1			
	Yes	2.492 (0.43–14.431)	0.308		
Alcoholic	No	1			
	Yes	0.096 (0.008–1.154)	0.065		
Age		1.094 (0.99–1.21)	0.078		
BMI		2.131 (1.335–3.403)	0.002	1.912 (1.346–2.716)	<0.001
DT		1.384 (0.984–1.947)	0.062		
VD		0.76 (0.583–0.989)	0.041	0.773 (0.633–0.943)	0.011
HbA1c		12.534 (3.808–41.253)	<0.001	9.082 (3.52–23.433)	<0.001
HDL-C		5.188 (0.407–66.127)	0.205		
LDL-C		1.636 (0.48–5.578)	0.432		
Chol T		1.118 (0.498–2.508)	0.787		
TG		22.431 (4.444–113.228)	<0.001	11.597 (3.267–41.164)	<0.001

OR: odds ratio; CI: confidence interval; FH: family history; CRC: colorectal cancer; BMI: body mass index; DT: duration of type 2 diabetes mellitus; VD: 25′ OH vitamin D3; HbA1c: glycated hemoglobin A1c; HDL-C: high-density lipoprotein cholesterol; LDL-C: low-density lipoprotein cholesterol; Chol T: total cholesterol; TC: triglyceride.

All factors that showed a significant association in the univariate analysis were included in the simple and multiple stepwise logistic regression analysis. *P* < 0.05 was considered statistically significant.

## 4 Discussion

Owing to upward socioeconomic mobility and progressive population aging, the prevalence of T2DM has shown a rapid increase throughout the world. At the same time, the incidence of malignant tumors, particularly those of the digestive system, is also raising rapidly. Several recent studies have shown that T2DM is an independent risk factor for cancers and affects the prognosis of the patients. CRC is one of the most common cancers of the digestive system. Epidemiological studies have shown that T2DM is an independent risk factor for CRC [18,19]. Detection of serum markers is a convenient method for CRC screening and facilitates early diagnosis of these patients.

In this study, we examined the risk factors associated with increased levels of CRC tumor markers. We found that BMI, VD, HbA1c and TG were independent risk factors. BMI is used as an index to quantify the relative body weight of an individual and categorize the person as low weight, normal, overweight or obese. Several recent studies have shown a strong association between BMI and cancer; thus, obesity is a risk factor for cancer, irrespective of the presence or absence of T2DM [20,21]. In addition to its regulatory effects in calcium and phosphorus metabolism, VD is also associated with cancer development. Epidemiological studies suggest that intake of calcium and VD can reduce the incidence of CRC by 20–30% [22]. High level of serum VD is associated with a low incidence of CRC, and this phenomenon is more pronounced in women over 60 years of age [23]. The molecular mechanism of this phenomenon may be related to the change in the expression levels of VD receptor (VDR) in tumor tissues. VDR belongs to the steroid hormone receptor superfamily and participates in many biological processes, such as cell proliferation, differentiation, apoptosis and immune response; significantly low expressions of VDR have been demonstrated in a variety of malignant tumors including CRC [24]. Through the role of VDR, VD can inhibit proliferation and induce apoptosis of cancer cells through a variety of molecular mechanisms, including via regulation of the activity of genes related to cell proliferation and differentiation, such as *p21*, *p27*, *c-myc*, *c-fos*, *c-jun*, *laminin* and *fibronectin* [25]. Inhibition of the activity of cyclin–cyclin-dependent kinase complex was shown to block the cell cycle [26]; upregulation of pro-apoptotic Bak expression was shown to induce cell apoptosis [27]. The results of our study are consistent with those of previous studies, in that the low level of VD was significantly correlated with increase in CRC-related tumor markers.

Diabetes increases the risk of tumorigenesis, and high HbA1c reflects poor glycemic control. Therefore, the results of this study and previous studies suggest that high HbA1c level is an independent risk factor for CRC [28]. Available evidence pertaining to the relationship between abnormal lipid metabolism and CRC is not consistent. Some studies suggest that elevated levels of TG increase the risk of CRC [29], while other studies have shown that TG is not associated with the incidence of CRC [30]. The inconsistent results may be attributable to the different sites of CRC. Previous studies have shown that left colon cancer is more related to environmental factors, while right colon cancer is more related to genetic factors; thus, the proportion of left and right colon cancer may affect the average blood lipid level [29]. In our study, high TG level was associated with increased level of CRC tumor markers; however, we did not perform a subgroup analysis.

There are some limitations in our research. As a retrospective study, the inherent bias of selection is inevitable. The relatively small sample size limits the statistical power of multivariate analysis. In this study, we analyzed the association between CRC serum markers and different variables. However, we are not sure whether these patients had CRC. Although the aim of this study attempts to explain that patients with T2DM should more frequently be tested for CRC serum markers, there is no clear conclusion as to how frequently should such population test for serum CRC markers. This needs to be studied later. All subjects in our study were residents of the Eastern region of China, which limits the representativeness of our findings. In addition, CRC is a cancer largely caused by environmental factors and lifestyle. Very few lifestyle-related variables were included in the analysis. However, statistical analysis still showed clinical significance. In this study, BMI, VD, HbA1c and TG levels were independent risk factors for increased CRC serum markers in patients with T2DM. Patients with increased BMI, HbA1c and TG as well as lower VD levels may benefit from more frequent screening for CRC tumor markers to facilitate early diagnosis of CRC.

## 5 Conclusion

In this retrospective case–control study, we examined the risk factors associated with increased levels of CRC tumor markers. We found that BMI, VD, HbA1c and TG were independent risk factors. Patients with increased BMI, HbA1c and TG and decreased VD levels may benefit from more frequent screening for CRC tumor markers to facilitate early diagnosis of CRC.
